# Nanostructures Control the Hepatocellular Responses to a Cytotoxic Agent “Cisplatin”

**DOI:** 10.1155/2015/925319

**Published:** 2015-07-12

**Authors:** Shimaa A. Abdellatef, Riho Tange, Takeshi Sato, Akihiko Ohi, Toshihide Nabatame, Akiyoshi Taniguchi

**Affiliations:** ^1^Cell-Materials Interaction Group, Biomaterials Unit, Nano-Life Field, International Center for Materials Nanoarchitectonics (MANA), National Institute for Materials Science, 1-1 Namiki, Tsukuba, Ibaraki 305-0044, Japan; ^2^Glycobiology Laboratory, Nagaoka University of Technology, 1603-1 Kamitomiokamachi, Nagaoka, Niigata 940-2137, Japan; ^3^MANA Foundry, International Center for Materials Nanoarchitectonics (MANA), National Institute for Materials Science, 1-1 Namiki, Tsukuba, Ibaraki 305-0044, Japan; ^4^Graduate School of Advanced Science and Engineering, Waseda University, 3-4-1 Okubo, Shinjuku-ku, Tokyo 169-8555, Japan

## Abstract

In drug discovery programs, the alteration between *in vivo* and *in vitro* cellular responses to drug represents one of the main challenges. Since the variation in the native extracellular matrix (ECM) between *in vivo* and 2D *in vitro* conditions is one of the key reasons for such discrepancies, thus the utilization of substrate that likely mimics ECM characteristics (topography, stiffness, and chemical composition) is needed to overcome such problem. Here, we investigated the role of substrate nanotopography as one of the major determinants of hepatic cellular responses to a chemotherapeutic agent “cisplatin.” We studied the substratum induced variations in cisplatin cytotoxicity; a higher cytotoxic response to cisplatin was observed for cells cultured on the nanopattern relative to a flat substrate. Moreover, the nanofeatures with grating shapes that mimic the topography of major ECM protein constituents (collagen) induced alterations in the cellular orientation and chromatin condensation compared to flat surfaces. Accordingly, the developments of biomimetic substrates with a particular topography could have potentials in drug development analyses to reflect more physiological mimicry conditions *in vitro*.

## 1. Background

The liver is considered as the largest internal organ of body; it is mainly composed of parenchyma cells or hepatocytes and a lesser number of stellar cells (fat storing cells), Kupffer cells (specific liver macrophage), and sinusoidal endothelial cells. The vitality of liver as an organ is based upon the metabolic, secretory, and detoxifying functions which are performed by the hepatocytes. Extracellular matrix (ECM) is the cellular native environment. It is composed of organic matters and macromolecules (i.e., collagens, laminins, fibronectin, glycosaminoglycans, and proteoglycans) that are naturally produced and assembled into three-dimensional networks with specifically contoured nanoarchitectures. Collagen is a fibrous protein that arranged into a triple helical structure with 300 nm in length and 1.5 nm in width [1]. These collagen helices are hierarchically assembled into networks of nanofibrils with diversified diameters (30–400 nm), lengths (up to tens of micrometers), and periodical cross-striations [2]. The architecture of ECM molecules possesses the required physical cues that trigger the optimum cellular behaviors [3–5]. Moreover, ECM is a crucial determinant of the compliance and functionality of liver despite its lower overall percentage. Thus, ECM provides the structural frameworks to maintain the hepatocytes states and related functionalities at the highest level.

Consequently, mimicking such ECM biophysical and chemical factors by the manipulation of biomaterial characteristics (i.e., topography, stiffness, roughness, surface chemistry, etc.) would participate in the generation of biospecialized hepatic systems for the drug development research [6] and tissue engineering applications [7]. Since any change in the native ECM characteristics (i.e., chemical composition or biophysical characteristics) will be recognized and transduced by cells through several receptors such as integrins (transmembrane proteins) into specific signaling pathways and provoke a variety of cellular cascades and events [8], the ECM chemical or physical characteristics in addition to signaling factors are responsible for continuous alterations in the growth, proliferation, tumor invasion, and transformation for a wide number of cell types [9–12]. The cellular response to drugs (toxicity/resistance) is proven to be changed by the alteration in ECM characteristics. Since several studies have been performed to clarify the relationship between naturally and artificially manipulated ECM characteristics and cellular responses to chemotherapeutic agents, for instance, the modifications of ECM compositions were accompanied by the alterations in the chemotherapeutic sensitivity [13, 14]. Another factor is the role of matrix stiffness in the alteration of cell responses to the chemotherapy. Since a stiffer artificial material was able to increase the therapeutic resistance with a lower clonogenic capacity, a softer material induced reversible cellular dormancy in hepatic cell lines [15]. For natural ECM, a decrease in the matrix stiffness by altering collagen crosslinking delayed the malignant growth and tumor development [16]. Moreover, the use of 3D porous polystyrene support as a substrate for cell cultures resulted in a lesser susceptibility to methotrexate cytotoxicity compared to 2D cultures [17]. Coculturing of hepatic cell line (HepG2) on 3D alginate-based hydrogels with MCF-7 was associated with an increase in the hepatic susceptibility and toxicity to chemical entities as acetaminophen and diclofenac compared to cells cultured in 2D flat surface [18]; these alterations in the cellular toxicities were well correlated with reported* in vivo* responses to these drugs [19]. The use of scaffolds fabricated from chitosan-alginate was associated with an increase in the malignancy and invasion of different glioma cells, since these scaffolds induced an increase in the production of mediators that could be necessary for angiogenesis [20]. Meanwhile, little attention has been paid to the role of nanotopography in the alteration of cellular responses to cytotoxic agents especially the nanotopography that mimics ECM topography, despite its importance in establishing an explicit relationship between single ECM characteristics and cellular responses to therapeutic agents.

In the present study, the alterations in the cellular response of a hepatic cell line under cytotoxic conditions were determined, while nanopattern substrates with various shapes (240 nm) were used as culture substrates to mimic the topography of an important ECM protein, in particular the dimensions and geometries of collagen molecules that assembled into fibrillar structures with a diameter range from 260 to 410 nm [21]. These substrates previously showed an improved cytocompatibility and hepatocellular functionality [22, 23]. Cisplatin is one of the most effective and widely used cytotoxic agents for the treatment of several types of cancers; it has a great affinity toward nucleophilic centers of biomolecules since it forms bifunctional adducts with such molecules. Despite the prevalent utilization of cisplatin in many kinds of cancer therapy, its associated side effects to other organs impede such utilization [24]. Therefore, the prediction of such associated side effects in a more biomimetic conditions* in vitro* could have great potentials. Accordingly, we investigated the alterations in the cellular cytotoxicity and morphology induced by culturing the cells on biomimetic substrates in the presence of cisplatin using fluorescent microscopy and scanning electron microscopy (SEM). We showed that the presence of these nanostructures which mimic ECM topography or biophysical characteristics was capable of affecting the hepatic cellular response to such cytotoxic agent. These results are important for understanding the relationships between ECM topography and cellular responses to chemotherapeutic entities.

## 2. Methods

### 2.1. TiO_2_ Nanopattern Substrates Fabrication Using Electron Beam Lithography and Atomic Layer Deposition

TiO_2_ nanopattern substrates were fabricated and characterized as previously reported [22]. A dry heat sterilization at 170°C for 1 hr was used for the sterilization of nanopattern and flat substrates.

### 2.2. Cell Culture

In a 100% humidified atmosphere containing 5% CO_2_ at 37°C, hepatic cell line (HepG2) was maintained in Dulbecco's MEM (DMEM, Nacalai Tesque, Kyoto, Japan) supplemented with 10% heat inactivated FBS (Biowest, Nuaille, France) and 100 U penicillin/100 *μ*g streptomycin (Nacalai Tesque, Kyoto, Japan) per each ml medium. At 70–80% confluency, cells were cultured over the nanopattern substrates for 8 hrs. Then cells were incubated with and without cisplatin solutions diluted in DMEM (at concentrations 0.25 mM, 0.5 mM, and 0.75 mM) for another 12 hrs (the total time for cells culture over nanopattern substrates was 20 hrs). A freshly prepared cisplatin (Sigma-Aldrich, St. Louis, MO, USA) in DMSO was used for each experiment (DMSO concentration in final medium dilution was less than 0.1%).

### 2.3. Live/Dead Viability Assay

HepG2 cells were cultured on nanopattern substrates and incubated with different concentrations of cisplatin were washed with PBS 3 times; then alive/dead viability kit (Invitrogen, Eugene, OR, USA) was used. The kit contains a nonfluorescent dye called calcein AM (1 *μ*M), which would be enzymatically hydrolyzed into a green fluorescent dye in living cells (excitation 495, emission 515), while the impermeable fluorescent dye ethidium-D-1 passes into dead cells and binds to nucleic acids to produce red fluorescence (excitation 595, emission 635). Observations were performed using an upright fluorescent microscope (Olympus BX51, Tokyo, Japan) equipped with an Olympus DP70 digital camera, and DP Controller Ver. 3.1.1 was used to process the images. The percentages of dead cells in a constant area (1.44 mm^2^) were calculated as the number of dead cells/total number of cells × 100; each experiment was repeated 3–5 times.

### 2.4. Scanning Electron Microscopy Investigation

SEM using (Hitachi S-4800, Tokyo, Japan) was used for the investigations of cellular alignment and morphological variations. After the cells were cultured on the substrates with and without cisplatin concentrations, the 2 hr incubation using 2.5 vol.% glutaraldehyde (Wako, Japan) in PBS was done for fixation and preservation of the cells morphology. Then, stepwise incubations with several of ethanol concentrations (10, 40, 60, 80, and 100%) for 5 min were used for the cell dehydration, respectively. After that, the freeze drying (Hitachi Es 2030, Tokyo, Japan) was done for desiccation of samples. Finally, the cells over nanopatterns were observed at an acceleration voltage in the range 1–5 kV.

### 2.5. Examination of Chromatin Condensation

HepG2 cells were cultured on nanopattern substrates and incubated with cisplatin, the cells were washed with PBS 3 times, and nuclear staining was done using Hoechst 33342 (Dojindo Molecular Technology Inc., Japan) at 5 *μ*g/mL and propidium iodide (Dojindo Molecular Technology Inc., Japan) at 1 *μ*g/mL for 5 min, followed by washing with PBS (2 times).

An upright fluorescence microscope (Olympus BX51) equipped with an Olympus DP70 digital camera was used for the observations of fluorescence staining using an. DP Controller Ver. 2.1.1 software (Olympus) was used to process the images.

Analysis of variance using one-way ANOVA and Fisher's LSD (Least significant difference) test for the determination of statistical significance using SigmaPlot 11.00 (Systat software Inc., Germany) were performed.

### 2.6. CTR-1 Immunofluorescence and Actin Fluorescence Staining

HepG2 cells were cultured on nanopattern substrates for 20 hrs; then the fixation step for the cells over nanopattern was performed using 4% paraformaldehyde (PFA) in PBS at 4°C for 15 minutes; afterward, the incubation with 0.1 M glycine for 5 min was done to neutralize any excess aldehyde. Then incubation with 1% Triton X-100 in PBS for 5 minutes was performed to increase the cell membrane permeability for the used antibodies. After that, a 1 hr incubation with polyclonal rabbit anti-CTR-1 diluted 1 : 1000 (Santa Cruz Biotechnology, Santa Cruz, CA, USA) was performed; then goat polyclonal anti-rabbit IgG-H&L-DyLight 488 diluted 1 : 500 (Abcam, UK) was added for additional 1 hr, and finally samples were observed using fluorescent microscopy. For the determination of cytoskeleton variations using F-actin staining, the cells were incubated with phalloidin-TRITC (Sigma-Aldrich, St. Louis, MO, USA) at 5 *μ*g/mL for 15 min.

## 3. Results

**Figure 1:**
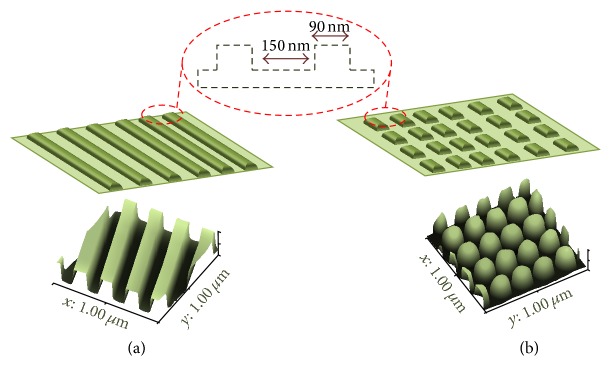
3D figures illustrate the geometrical characteristics using AFM analysis for (a) gratings and (b) rectangles nanopatterns, respectively, with an illustration of the size and interspace dimensions between these nanostructures.

In this study, the role of substrate nanotopography as a major determinant of hepatocellular cytotoxic responses over a short time of exposure was reported on which nanopatterns with various geometries were utilized as cultured substrates for the hepatic cell line (HepG2). As these assembled constructs were expected to closely mimic the topography of the native ECM protein (collagen)* in vivo*, such nanopattern substrates were fabricated using electron beam lithography and atomic layer deposition as previously reported [22]. The shape and dimension of fabricated nanopattern are illustrated using AFM and SEM (Figure 1).

### 3.1. Alteration in Cisplatin Cytotoxic Responses

**Figure 2:**
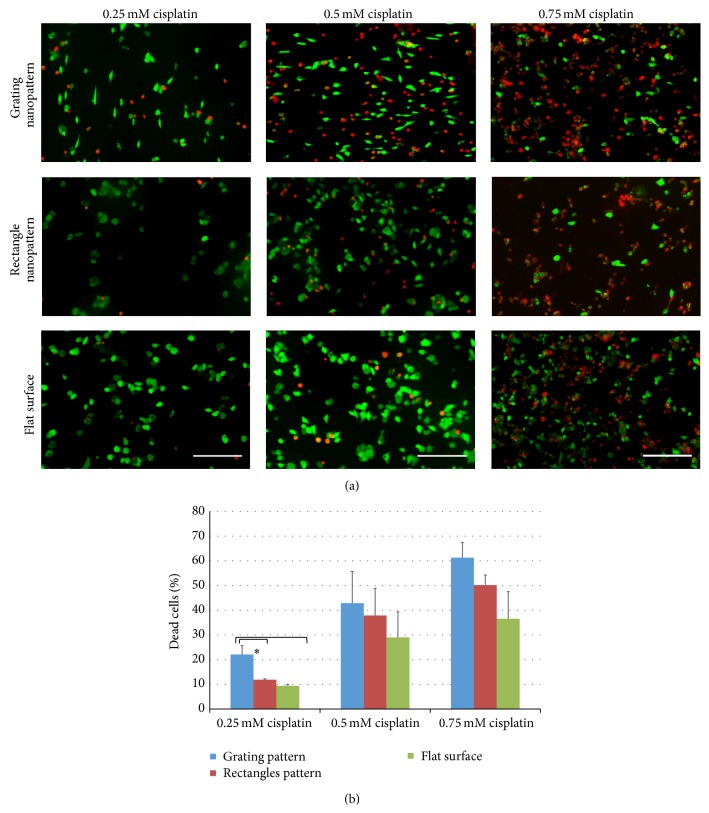
(a) Live/dead fluorescence staining of HepG2 cells cultured on the diversified geometry of nanopattern (grating and rectangles) and flat surface, on which green cells are alive cells and red cells are dead cells. (b) Average percentages of dead cells (*n* ≥ 3) upon culturing HepG2 on different substrates with various cisplatin concentrations. ^*∗*^Statistically significant at *P* < 0.05. Scale bar = 200 *μ*m.

Here, we examined the influence of substrates' nanotopography with various geometries on cisplatin cytotoxic responses. An increase in the percentage of dead cells was observed after culturing HepG2 cells over the nanopattern substrates compared to a flat surface after exposure to various cisplatin concentrations for 12 hrs.  Figure 2 shows fluorescent micrographs of dead (red) and alive (green) HepG2 cells cultured on gratings/rectangles nanopattern substrates and compared to flat surfaces in the presence of three different concentrations of cisplatin. In general, the biocompatibility of such substrate was previously reported [22, 23], as there is no change in the viability of cells cultured on the substrates alone without any cytotoxic agent. However, after cisplatin exposure, the modulation of topography significantly influenced the cytotoxicity induced by cisplatin (red fluorescent cells).

A significantly higher cytotoxic response was observed for the nanopattern with grating shape relative to the flat surface (Figure 2(a)). The percentages of dead cells showed nearly 2-fold increases upon the change of topography to grating shape, especially with 0.25 mM cisplatin concentration relative to the flat surface (*P* < 0.05) (Figure 2(b)), while in such cisplatin concentration the change in geometries of substrate topography from a grating shape into a rectangle shape resulted in a significant reduction in such percentage. Meanwhile, the culture of HepG2 over nanopattern substrates with gratings shape in the highest cisplatin concentration (0.5 mM and 0.75 mM) showed an increase in the percentage dead cells, but it was not statistically significant compared to the flat surface using such statistical analysis. Furthermore, the percentage of dead cells cultured on the rectangle shape nanopattern showed an increase compared to flat surface at various cisplatin concentrations; however, such increase was statistically insignificant. These results emphasize the influence of nanotopography as a modulator of cellular responses to a cytotoxic agent.

### 3.2. Hepatocellular Nuclear and Morphological Variations

The cellular exposure to cisplatin induces specific cellular and morphological variations. Meanwhile, the change in the substrate topography alone induces transformations in the shape, cellular orientation, cell alignment, and elongation. Therefore, we examined these cellular variations induced by cisplatin in the presence of such biomimetic constructs. Thus, the morphological variability, directional modifications, and nuclear chromatin condensation were examined by different techniques such as fluorescent microscopy and SEM.

#### 3.2.1. Morphology, Cellular Alignment, and Orientation

**Figure 3:**
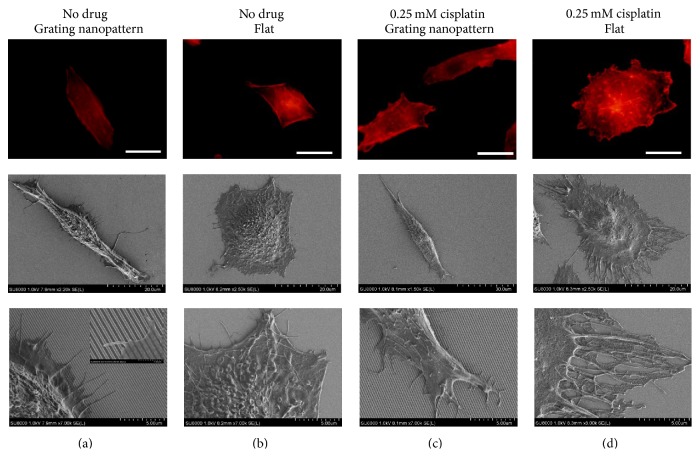
Actin filament rearrangement and morphological alteration with subsequent cellular alignment parallel to nanogratings upon culturing of cells on the nanopattern (a) or a flat surface (b) without any drug and gratings nanopattern (c) or a flat surface (d) with 0.25 mM cisplatin (scale bar = 20 *μ*m).

HepG2 cultured on the grating nanopattern substrate without cisplatin displayed a fundamentally different morphology compared to cells cultured on the flat surface. The cellular alignment in the longitudinal direction parallel to the nanogratings was maintained with an alteration in cellular spreading. Furthermore, the parallel rearrangements of actin filaments to the nanograting axes were observed. However, cells cultured on flat surfaces did not show such alignment (Figures 3(a) and 3(b)). Moreover, filopodia fixations as anchoring points in the planer area between such nanostructures were observed by SEM (Figure 3(a), inset). In the presence of a low cisplatin concentration (0.25 mM), further actin polymerization and remodeling were observed. The maintenance of topography-induced parallel cellular alignment was observed (Figure 3(c)) with subsequent formation of orderly arranged filopodia for cells cultured over the grating nanopattern substrates with the cytotoxic agent. Furthermore, the appearance of small blebs due to apoptosis on the cellular interface was observed. Meanwhile, for the cells cultured over the flat surface, cisplatin induced the reorganization of actin filaments with remodeling especially in the cellular periphery (Figure 3(d)).

**Figure 4:**
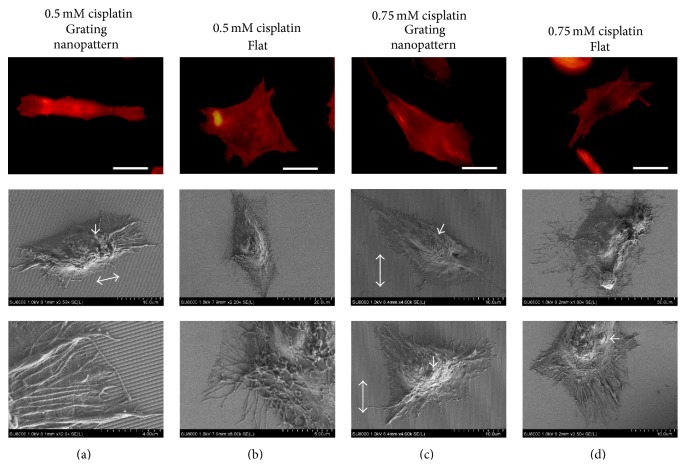
Morphological alteration induced by culturing HepG2 cells over grating nanopattern (a) and flat (b) substrate with 0.5 mm cisplatin concentration and grating nanopattern (c) and flat (d) substrate with 0.75 mm cisplatin (for fluorescent micrographs' scale bar = 20 *μ*m). The formations of excessive cytoplasmic extensions and irregularities were observed with actin rearrangements followed by subsequent cellular misalignment to the nanogratings at higher concentrations of cisplatin (0.75 mM). Double arrows represent the directions of the grating nanopattern, and arrows show blebs formed as a result of apoptosis.

In a higher cisplatin concentration such as 0.5 mM, the formation of irregular cytoplasmic ledges was observed with an increase in the number of blebbing cells (Figure 4(a)), while the cellular alignment and orientation parallel to the grating nanopattern were maintained with their exploring filopodia (Figure 4(a)). However, at this concentration (0.5 mM), the flat substrate maintained actin filament reorganization with immoderate cytoplasmic treelike extensions (Figure 4(b)) and morphological cellular anomalies. Furthermore, with the use of 0.75 mM cisplatin, the cells cultured over the nanopattern substrates started to lose their nanograting recognition capabilities with subsequent loss of cellular alignment and parallel orientations (Figure 4(c)). However, at this concentration, cells cultured on flat surfaces maintained their eccentric cytoplasmic contoured irregularities with the formation of large blebs (Figure 4(d)).

#### 3.2.2. Alterations in Chromatin Condensation

**Figure 5:**
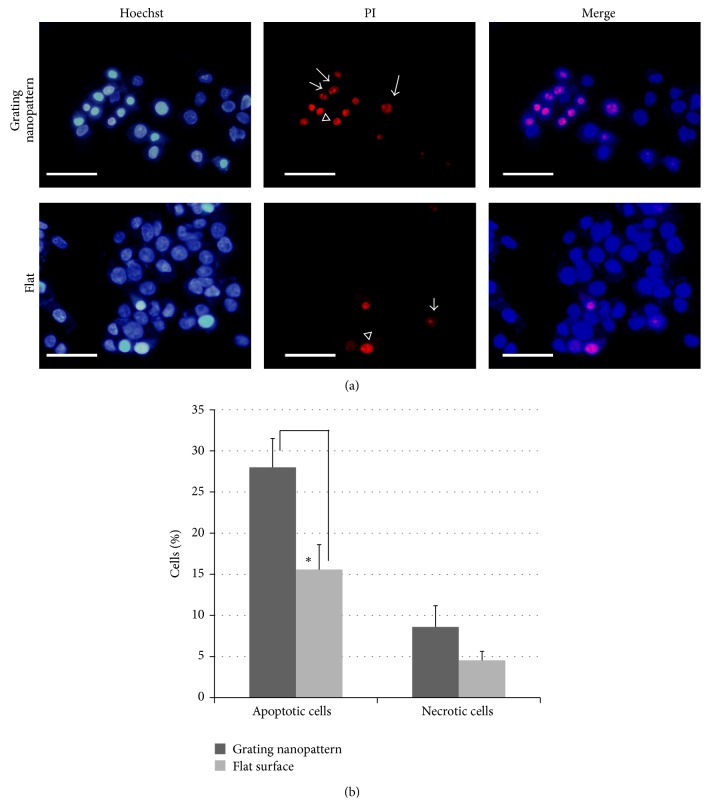
(a) Fluorescent staining of HepG2 cellular nuclei cultured on grating nanopattern (upper panel) and a flat surfaces (lower panel). Arrows show phenotype I (apoptotic cells), and arrowheads show phenotype II (necrotic cells) (scale bar = 50 *μ*m). (b) Alteration in number of cells cultured on various substrates showing different phenotypes of chromatin condensation induced by the presence of cisplatin. The percentage of apoptotic phenotype induced by cisplatin was significantly higher in cultures on the grating nanopattern substrate. ^*∗*^ *P* < 0.001.

To investigate if the increase in the percentage of dead cells was related to necrosis or apoptosis, we have examined the nuclear alterations related to apoptosis after cisplatin exposure. Fluorescent staining of HepG2 nuclei with Hoechst (blue) and PI (red) and the percentages of these cells cultured on various substrates were illustrated in Figure 5. Since the presence of cisplatin induces different chromatin condensations in HepG2 cells compared to controls (no drug), the grating nanopattern substrates stimulate significantly (2-fold increase) chromatin condensations for apoptotic cells (a specific nuclear phenotype) relative to the flat surface (*P* < 0.001) (Figure 5(b)).

### 3.3. Role of CTR-1 Expression

**Figure 6:**
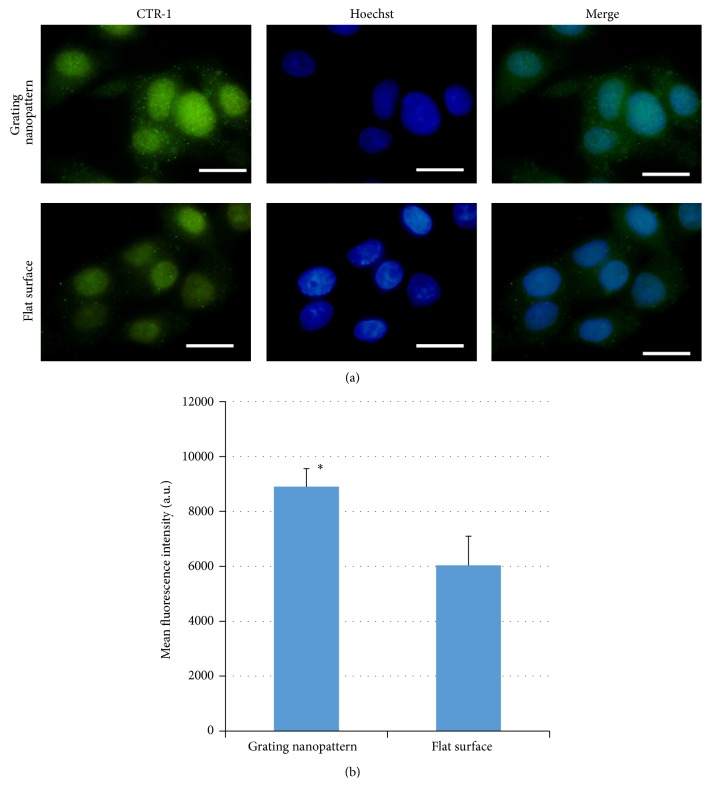
(a) Immunofluorescent staining of copper transporter-1 (green excitation *λ* = 448 nm) in HepG2 cells cultured on grating nanopattern with total dimensions (240 nm) and grating shapes (upper panel) and flat surface (lower panel) (scale bar = 20 *μ*m). (b) Calculated mean fluorescence intensity that shows a significant increase in the fluorescence intensity for cells cultured over grating nanopattern substrate compared to flat surface. ^*∗*^Statistically significant at (*P* < 0.05).

Here, we examined the expression of one important protein that alters cisplatin uptake and associated cytotoxicity which is copper transporter-1 (CTR-1) using immunofluorescence staining. As such protein has a key role in the initial influx and active transport of cisplatin and other platinum drugs.  Figure 6 shows fluorescent images of CTR-1 upon culturing of HepG2 on grating nanopattern and flat substrates. The expression CTR-1 had almost 1.3-fold increase after culturing of HepG2 on the 240 nm linear nanopattern (Figure 6(a), upper panel) for 20 hrs (equivalent to the total duration for which HepG2 cells cultured on nanopattern/flat substrates in dead/alive experiment) compared to cells cultured on flat surface (Figure 6(a), lower panel). Such increase in CTR-1 expression was statistically significant (Figure 6(b)). Thus, the presence of specific nanostructures that mimics ECM topography likely stimulated the production of CTR-1 compared to cells cultured on flat surface. This increase in CTR-1 production may be one of expected reasons for the observed alteration in cellular responses to cisplatin.

## 4. Discussion

Topography is one of important ECM physical characteristics that alter the cellular responses* in vivo*, since ECM topography is responsible for the activation of intricate networks of signaling pathways through extensive transmembrane receptor (i.e., integrins). These receptors transfer extracellular mechanical signals into altered cellular responses by the activation of several signaling pathways and proteins such as MAPKs (mitogen activated kinases) [25]. We cannot override the presence of other important cellular and ECM related characteristics such as chemical factors (presence of specific motifs, peptide, proteins, and growth factors) or the coexistence of diversified cells in several arrangements that would play important roles in the alteration of cellular responses to various drugs and biopharmaceuticals. In this study, electron beam lithography and atomic layer deposition were used to fabricate well-defined nanostructures with 240 nm lateral dimension and various geometries. Such nanostructures' size and geometry are in a close resemblance to the topography of human liver collagen nanofibrilar that arranged in the form of bundles with diameter range from 0.2 *μ*m to 2 *μ*m [26]. Furthermore, a higher cytotoxic response was observed for hepatic cells cultured on the gratings nanopattern relative to rectangle shape substrates or flat surfaces; these suggest the role for artificial nanotopography that closely mimics native ECM topography as a key player in the determination of cellular responses to cytotoxic agents and drugs. While the utilization of rectangles nanofeatures that less likely mimic such collagen fibrils resulted in a decrease in the observed sensitivity to cisplatin, indeed, the underlying mechanisms for such observations need further studies at the molecular level to understand the influence of such topography at cellular responses.

Cell morphology in general and the rearrangement of actin filaments in particular are major determinants of cellular behavior. Actin is a major cytoskeletal protein that polymerizes within cells to form thin, flexible fibers (filaments) approximately 7 nm in diameter and up to several micrometers in length. These filaments are organized into highly ordered structures, forming bundles or three-dimensional networks. The assembly and disassembly of actin filaments provide mechanical support, determine cell shape, and finally allow for movement of the cell surface, migration, and endocytosis. Furthermore, the continuous remodeling and polymerization of actin filaments play a key role in oncogenic signaling pathways [27], while here, we showed that the morphological changes associated with cisplatin exposure were modified due to the alteration in the substrate topography to likely mimic the ECM. These concentration-dependent alterations include changes in surface characteristics, cellular shape, and alignment. Consequently, these observations underline the role of the substrates nanotopography as one of the significant ECM characteristics in the determination of morphological and cellular responses to a drug.

The cellular toxicity to cisplatin is associated with apoptosis and the formation of specific nuclear rearrangements and chromatin condensation [28]. Such morphological variations could be observed and differentiated using fluorescence staining of nuclei with Hoechst and propidium iodide (PI), which allowed the differentiation of apoptosis and necrosis. Since necrotic cells will have cells with large nuclei intensely stained with Hoechst and PI, apoptotic cells are double-stained with Hoechst with condensed nuclei with PI-stained spots [29]. The cells with explicit signs of apoptosis such as a decrease in cellular size and chromatin condensation with subsequent localization in nuclear ledges were significantly increased (Figure 5(a)). However, the percentage of the other nuclear phenotype (necrosis) stimulated by cisplatin did not show any significant increase upon culturing HepG2 cells over the grating nanopattern compared to the flat substrate (Figure 5(b)). These results emphasize that cisplatin induced apoptosis was increased on the grating nanopattern substrates compared to flat surface; in other words, the grating nanopattern that mimics the ECM physical topography aggravated the cisplatin cytotoxicity.

Even though the exact underlying mechanisms of such varied cellular responses are still unknown, we can consider the alteration of four possibilities: (1) cisplatin uptake, (2) intracellular signaling, (3) intracellular efflux of cisplatin, and (4) inactivation of cisplatin. Thus, any change induced by substrate's nanotopography affecting any of these factors could be responsible for the altered cytotoxic response. The manipulation of cisplatin uptake is a tempting explanation for our observations. The uptake of cisplatin is regulated by a variety of mechanisms [30, 31] that include passive diffusion, endocytosis, and CTR-1 receptors. Copper transporter-1 (CTR-1) is one of the strong candidates, as it has a key role in the initial influx and active transport of cisplatin [32]. CTR-1 is a channel-like transporter membrane protein with 3 transmembrane domains [33]. Alterations in cisplatin uptake have been associated with the presence or absence of these receptors, and the deletion of CTR-1 from fibroblasts is responsible for an 80% reduction in cisplatin uptake [34]. Furthermore, CTR-1-overexpressing cells have significantly increased intracellular accumulation of cisplatin [35]. We observed that the level of CTR-1 is significantly modulated by the mimicking of natural ECM topography induced by the 240 nm grating nanopattern. We cannot conclude that the observed increase in CTR-1 expression is the only reason for the topography-induced cytotoxic response. However, we can suggest that it plays a role in such observations as the underlying mechanisms for the topography-induced alteration in the observed cellular responses are not fully clear and need further exploration in the future studies. Furthermore, recently, the endocytosis of small molecular weight chemicals and DNA was previously reported to be modified by substrates micro- and nanotopography* in vitro* [36]. Such increase in the cellular uptake was explained by the topography-induced alteration in the formation of densely packed actin filaments which affected the cellular contractility and upregulation of the Rho-GTPase pathways. Concomitantly, several morphological changes induced by topography were responsible for the induction of various cellular responses other than endocytosis as previously reported in the literatures [37–41].

## 5. Conclusions

In summary, we examined the hepatic cell responses to cisplatin in the presence of manipulated substrates that mimic one of ECM topography* in vitro*. Since the modulation of topography greatly increased the cytotoxicity induced by cisplatin, a higher cytotoxic response was observed when cells were cultured over the gratings nanopattern relative to flat surfaces. Furthermore, cellular responses induced by the altered substratum topography were geometry specific. Additionally, in the presence of cisplatin, the grating nanopattern induced a transformation in the cellular shape, orientation, cell alignment, and chromatin condensation relative to a flat surface. Thus, we conclude the important role of substrate physical characteristics as topography in the determination of hepatocellular responses to a cytotoxic agent. A deep understanding of this role for a wide array of cells could be further exploited to produce distinguished biomimetic surfaces. Such surface could be utilized for the development of more accurate and sensitive drug toxicity analysis* in vitro*.
